# Bacterial identification in clinical samples – comparing Nanopore and Illumina sequencing of the 16S rRNA and *rpoB* genes

**DOI:** 10.1186/s12879-026-14101-z

**Published:** 2026-07-28

**Authors:** Joanna Małgorzata Bivand, Audun Sivertsen, Marit Gjerde Tellevik, Oystein Saebo, Robin Patel, Ruben Dyrhovden, Øyvind Kommedal

**Affiliations:** 1 Department of Microbiology, Haukeland University Hospital, Bergen, Norway; 2 Department of Clinical Science, University of Bergen, Bergen, Norway; 3 Pathogenomix, Santa Cruz, CA USA; 4 Division of Clinical Microbiology, Department of Laboratory Medicine and Pathology, Division of Public Health, Infectious Diseases, and Occupational Medicine, Department of Medicine, Mayo Clinic, Rochester, MN USA; 5 Department of Medicine, Haukeland University Hospital, Bergen, Norway

**Keywords:** Nanopore sequencing, 16S rRNA gene, *rpoB* gene, Illumina, Bacterial identification, Clinical microbiology

## Abstract

**Background:**

Recent improvements in the sequencing accuracy of the Oxford Nanopore Technologies (ONT) platform have increased its suitability for clinical microbiology applications, including broad-range amplification and sequencing of the 16S ribosomal RNA (rRNA) gene directly from clinical samples. This approach has a strong potential to improve diagnosis of bacterial infections. However, even full-length 16S rRNA sequencing may lack sufficient resolution to distinguish closely related species, a limitation exacerbated by the remaining ONT error rate. The *rpoB* gene represents a high-resolution bacterial phylogenetic marker that offers improved species-level discrimination. We evaluated a strategy combining ONT sequencing of the V1-V3 segment of the 16S rRNA gene and a segment of the *rpoB* gene. In addition, we assessed two novel pipelines designed specifically for the analysis of ONT amplicon data in clinical microbiology, where knowing the associated confidence level for a given identification is crucial, as is the ability to detect organisms that may not be represented in reference databases.

**Methods:**

A staggered mock community and 25 abdominal abscess samples were investigated using ONT sequencing of the 16S rRNA and *rpoB* genes, with Illumina sequencing of the same genes used as a comparator method to validate the analytical accuracy of the approach.

**Results:**

*rpoB* ONT sequencing achieved species-level identification in 91.2% of detections, as compared to 62.8% with 16S rRNA ONT sequencing. However, 16S rRNA ONT sequencing obtained a higher sensitivity of 93%, as compared to 83.7% for *rpoB* ONT sequencing. Combining 16S rRNA and *rpoB* ONT sequencing yielded a sensitivity of 98.8% with 89% species-level discrimination. For both gene targets, ONT sequencing paralleled Illumina sequencing.

**Conclusions:**

Combining ONT sequencing with high-sensitivity broad-range amplification of the 16S rRNA gene and amplification of the *rpoB* gene for enhanced taxonomic resolution enables accurate detection and species-level identification of bacteria directly from clinical samples. This approach represents a rapid, flexible and cost-efficient tool for culture-independent diagnostics in clinical microbiology.

**Supplementary Information:**

The online version contains supplementary material available at https://doi.org/10.1186/s12879-026-14101-z.

## Background

Broad-range amplification of the bacterial 16S ribosomal RNA (rRNA) gene directly from clinical samples followed by next-generation sequencing has highlighted shortcomings of bacterial culture, particularly for samples containing anaerobic or fastidious microbes and for those from patients who have received antibiotics prior to sample collection [1–5]. Despite its potential, the adoption of 16S rRNA targeted next-generation sequencing (16S TNGS) in clinical microbiology has been slow, mainly due to high cost and long turn-around time. Oxford Nanopore Technologies (ONT) sequencing is a rapid, long-read sequencing technology offering smaller, single-use flow cells (Flongle flow cells) with a cost and capacity potentially suitable for analysis of a single sample. A higher sequencing error rate and lack of appropriate bioinformatics tools have prevented widespread adoption of this platform in diagnostic microbiology. Today, with a read accuracy exceeding 99% combined with dedicated bioinformatics approaches, ONT sequencing is beginning to yield results of sufficient quality for infectious disease diagnostics [6–8].

A limitation of 16S targeted sequencing is poor species-level resolution within some clinically important genera [9]. This problem is exacerbated by the increasingly fine-grained bacterial taxonomy, where novel species are often the result of splitting of previous polyphyletic taxa into two or more closely related species [10–12]. Even full-length 16S rRNA gene sequencing will not uniformly resolve these challenges, and the actual improvement in classification as compared to the partial 16S rRNA V1-V3 region is debatable [6, 8, 13, 14]. *rpoB* is an alternative marker gene with significantly better species-level resolution [15–17]. Recently, we described the first broad-range *rpoB* primers suitable for bacterial detection and identification in clinical microbiology [18]. The resulting ~ 550 bp (bp) amplicon is well-suited for ONT sequencing.

According to CLSI guidelines for bacterial identification based on the 16S rRNA gene, unambiguous species-level identification generally requires ≥ 99% homology with a high-quality reference and a distance to the next alternative species of > 0.8% [19]. Similar criteria have been published for *rpoB*, recommending a homology to a high-quality reference of ≥ 98.5% and a distance to the next alternative species > 1.4% [18]. Such criteria reflect the phylogenetic properties and biological variation of the respective genes and should be used regardless of sequencing method. However, their application is dependent upon an alignment and cannot be coupled with pure k-mer based identifications. Alternative more suitable bioinformatic approaches combine k-mer based binning of similar reads with subsequent consensus sequence generation from each bin before final alignment-based identifications using BLAST. Additionally, the use of public databases presents several challenges, including taxonomic misannotations, different taxonomic classifications between databases, or underrepresentation of some genera [20]. These issues affect the performance of bioinformatics tools, which need to be designed to detect all bacteria, even those that are not present in the databases. In clinical microbiology, this aspect is essentially important, as every species may be clinically relevant and have a direct impact on how a particular infection is treated.

In the present study, we investigated the performance of targeted ONT sequencing of partial 16S rRNA and *rpoB* genes for bacterial detection and identification directly from clinical samples, with Illumina sequencing of the same target genes as the comparator method. The use of two gene targets, two sequencing methods and three bioinformatics pipelines, allowed for a thorough evaluation of the analytical accuracy of proposed approaches.

## Methods

### The aim, design and setting of the study

This feasibility study was conducted at the Department of Microbiology, Haukeland University Hospital, Bergen, and assessed ONT amplicon sequencing of two gene targets, the 16S rRNA and *rpoB*, for bacterial detection and identification from clinical samples. The investigation included (i) ONT sequencing of partial *rpoB* (550 bp) and 16S rRNA amplicons from 25 intraabdominal samples and a commercial staggered mock community; (ii) design of a novel broad-range *rpoB* forward primer to pair with our previously published broad-range *rpoB* reverse primer, generating a shorter ∼450 bp amplicon suitable for Illumina sequencing; (iii) Illumina sequencing of partial *rpoB* and 16S rRNA amplicons from the same samples; (iv) comparison of results obtained by ONT with those from the established Illumina pipeline; (v) design and evaluation of a new in-house pipeline for analysis of ONT sequencing data; and (vi) assessment of the diagnostic value of *rpoB* compared to 16S rRNA gene sequencing for the Illumina and ONT sequencing platforms.

### Biological samples and controls

Remnant extracted DNA from 25 abdominal abscess samples, previously investigated with 16S rRNA gene PCR and Sanger sequencing in routine clinical practice, were studied. Extraction of bacterial DNA was performed as described previously [21]; the extracted DNA had been stored at -80 °C at the Department of Microbiology, Haukeland University Hospital, Bergen, Norway.

A commercial mock community (ZymoBIOMICS^®^ Gut Microbiome Standard, Microbiomics, Zymo Research, Irvine, California, USA) was used to evaluate the efficiency and accuracy of the proposed approaches. The commercial mock community was diluted 1:100 in nuclease-free water. The dilution was further diluted 1:2 in MagNa Pure Bacterial Lysis Buffer (BLB) (Roche Diagnostics, Mannheim, Germany) before mechanical lysis of bacterial cells in a Lysing Matrix E bead-containing tube (MP Biomedicals, Santa Ana, CA, USA) and run two times at 45s with speed 6.5 m/s in MagNA Lyser instrument (Roche Diagnostics, Mannheim, Germany). Extraction of bacterial DNA was performed using the ELITe InGenius automated extractor with the ELITe InGenius SP 200 Extraction Kit (ELITechGroup MDx, Turin, Italy) following the manufacturer’s protocol.

For ONT sequencing, negative controls consisting of BLB and PCR-grade water were extracted together with clinical samples and included with each PCR setup. Seven extraction controls for the 16S rRNA gene and four extraction controls for *rpoB* were included in the PCR runs for the clinical samples and sequenced separately on separate Flongle flow cells.

For Illumina sequencing, positive (*Yersinia enterocolitica* ATCC 23715) and negative (BLB and PCR-grade water) extraction controls were included with each sequencing run.

### PCR primers

Primers are listed in Table 1. The *rpoB* primers and 16S V1-V3 primers used for ONT sequencing have been described and evaluated previously [18, 21, 22].

The 16S rRNA primers used for Illumina sequencing are those recommended in the standard Illumina protocol [23], with modifications as described previously [22]. For the design and evaluation of the novel Illumina *rpoB* forward primer, reference sequences from the NCBI database were aligned in Geneious bioinformatics software version 9.1.7 (Dotmatics, Auckland, New Zealand). Combined with our previously described *rpoB* reverse primer [18], the resulting Illumina *rpoB* amplicon corresponded to *rpoB* nucleotide positions 1,675 to 2,068 of the *Escherichia coli* ATCC 11775 type strain, with amplicon sizes ranging from 388 bp to 445 bp depending on the species. Universality of the primer was confirmed in silico against bacteria representing 124 clinically relevant genera. In addition, as a supplementary evaluation of the coverage of *rpoB* primers, an extensive in silico PCR analysis was conducted using the Usearch11 software (https://github.com/rcedgar/usearch_old_binaries*)* [24] and the Genome Taxonomy Database (GTDB). Mismatches in primer 3´-end are believed to impair amplification, and other significant mismatches for clinically relevant species are displayed in Supplementary Table 1. Most mismatches were against members of the normal human microbiota, only rarely involved in infections relevant for broad-range amplification and detection. The clinically most relevant mismatches were for *Bilophila wadsworthia*, the *Lactobacillus gasseri* complex and *Ureaplasma* spp.

**Table 1:** 16S rRNA and *rpoB* primers for ONT and Illumina sequencing. In the primer sequence, M is A or C; Y is C or T; R is A or G; H is A, C, or T; W is A or T; D is A, G, or T; K is G or T; N is any base; I is deoxyinosine; underlined letters indicate a modified locked nucleic acid (LNA-base)

Sequencing method	Primer names	Sequence	Study
16S ONT	16S_DPO_Short-F 16S_DPO_Short-R	5´-AGAGTTTGATCMTGGCTCAIIIIIAACGCT-3´ 5´-CGGCTGCTGGCAIIIAITTRGC-3´	Kommedal et al. [21], Dyrhovden et al. [22]
16S Illumina	16S-F 340 F 16S-R 784R	5´-CCTACGGGNGGCWGCAG-3´ 5´-GACTACCAGGGTATCTAAKCC-3´	Illumina [23], Dyrhovden et al. [22]
*rpoB* ONT	RpoB_DPO1-F RpoB_DPO2-F RpoB_DPO-R	5´−TCNCARTTYATGGAYCAIIHIAAYCC − 3´ 5´−AGNCARTTYATGGAYCAIIHIAAYCC − 3´ 5´-CNGCYTGDCKYTKCATRTTIIIICCCAT-3´	Bivand et al. [18]
*rpoB* Illumina	RpoB_Illumina_DPO-F RpoB_DPO-R	5´-TGYCCIRTHGARWCICCIGARGGICIIAAYAT-3´ 5´-CNGCYTGDCKYTKCATRTTIIIICCCAT-3´	This study Bivand et al. [18]

### Amplification of partial 16S rRNA gene and *rpoB*

The real-time PCR assays were carried out using a QuantStudio™5 Real-Time PCR System (Applied Biosystems). The 16S rRNA and *rpoB* PCRs for ONT sequencing were run as previously described [18]. For Illumina sequencing, amplification of the 16S rRNA V3-V4 region was performed as previously described [21]. The Illumina *rpoB* PCR mixture (25 µl) contained 12.5 µl TB Green Premix ExTaq (Takara Bio, Kusatsu, Japan), 4 µl forward primer, 3 µl reverse primer (both from 10 µM solutions), 3.5 µl PCR-grade water, and 2 µl of template DNA. The PCR thermal profile included an initial denaturation at 95 °C for 30 s, 45 cycles of denaturation at 95 °C for 20 s, annealing at 50 °C for 20 s, and elongation at 72 °C for 30 s.

### ONT sequencing

Library preparation was performed using the Ligation Sequencing Kit V14 (SQK-LSK114) and the Ligation Sequencing Amplicons V14 protocol (ONT).

Samples were sequenced on the GridION platform using R10.4.1 Flongle flow cell for 6 h with super-accurate basecalling and minimum Q-score of 10.

### Illumina sequencing

The library was prepared according to the Illumina 16S Metagenomic Sequencing Library Preparation protocol [23], with minor modifications [22]. Sequencing was performed using the MiSeq Reagent Kit v3 (2 × 300 bp) on the Illumina MiSeq System (Illumina, Redwood City, CA).

### Taxonomy criteria

For taxonomic assignments, three classification levels were used: (1) Species-level: ≥99% (16S)/≥98.5% (*rpoB*) identity with a reference sequence, combined with a ≥ 0.8% (16S)/≥1.4% (*rpoB*) distance to the next closest species. (2) Species-group: identification meeting the species-level homology threshold, but with a distance to the next closest species below 0.8% (16S) or 1.4% (*rpoB*). (3) Genus-level: when the homology score was below the species-level criteria but ≥ 97%.

### Contaminant filtering and reference standard

For contaminant filtering, the sample-specific cut-off criteria described by Dyrhovden et al. [25] were applied. Filtered contaminants and detections in negative controls are listed in Supplementary Tables 2 and Supplementary Table 3.

For the clinical samples, a composite reference standard was used: non-contaminant detections made by at least two methods were considered confirmed detections. All non-contaminant detections made by only one method were considered as unconfirmed.

### Bioinformatic software and pipelines

As the primary analysis tool for this study, we used the RipSeq commercial software (Pathogenomix, Santa Cruz, CA). The RipSeq software includes one module for analysis of Illumina/IonTorrent amplicon data and a separate module for analysis of ONT amplicon data. The two modules are based on different bioinformatic approaches. In addition, we developed a new in-house pipeline, “Flexible Joint Operational pipeline for Reference-based Diagnostics (FJORD)” for ONT amplicon data analysis, which employs open-source tools and methods to achieve similar accuracy as the RipSeq software.

### Reference databases

The primary RipSeq 16S rRNA reference database was the “Pathogenomix Prime 16S” (Pathogenomix) database containing 3000 manually curated complete gene references, supplemented with (i)16S rRNA references extracted from all whole genome references in GenBank; (ii) GenBank 16S rRNA references linked to a scientific publication; (iii) all 16S rRNA references included in the “The All Species Living Tree” Project (https://imedea.uib-csic.es/mmg/ltp/*);* and (iv) all 16S rRNA references from the Human Oral Microbiome database (https://homd.org/*).* The RipSeq *rpoB* database and *rpoB* database used in the in-house pipeline were derived from the GTDB database (https://gtdb.ecogenomic.org/*)* [26]. The 16S rRNA database used in the in-house pipeline was constructed from the SATIVA-curated 16S GTDB database [27]. A detailed description of database construction is provided in Supplementary Material 1. GTDB has implemented a standardized microbial taxonomy based on genome phylogeny. The GTDB taxonomy uses reference strains as fixed points for nomenclature and classification, and Average Nucleotide Identity (ANI) distance (> 95%) of circumscribed species to representative genomes. This results in clusters forming around type strain genomes with > 95% ANI. Unsuffixed names represent clusters or lineages that contain a type strain, whereas an alphabetic suffix at the end of a species name indicates a placeholder name for a cluster without a type strain, suggestive of a novel, undescribed species [28].

### Sequence analysis using RipSeq - Illumina

FASTQ-files were individually analyzed using the NGS module in the RipSeq software (Pathogenomix) [1, 4, 22]. Briefly, sequencing reads shorter than 300 bp and any remnant primer sequences were removed before *de novo* clustering of reads into operational taxonomic units (OTUs) using homology thresholds of 99.0% for the 16S rRNA gene and 98.5% for *rpoB*. The most representative sequence from each OTU was subsequently identified using a standard BLAST search against the “Pathogenomix Prime 16S” database or the novel GTDB-based *rpoB* database, allowing for implementation of CLSI criteria for valid species-level assignments (supplemented with searches against the relevant NCBI GenBank database if only a low homology score was obtained with the standard databases).

### Sequence analysis using RipSeq – ONT

FASTQ files were analyzed individually using the novel Long Read Sequencing (LRS) module optimized for ONT in the RipSeq software. Reads shorter than 450 bp for the 16S rRNA gene or 500 bp for *rpoB*, and any remnant primer sequences were trimmed before k-mer based read binning against the reference-databases for the 16S rRNA gene (GenBank RefSeq 16S database) or *rpoB* (the novel GTDB-based database). Subsequently, consensus sequences were generated based on multiple alignments of reads that had binned to the same reference (consensus sequence generation). Finally, consensus 16S rRNA gene and *rpoB* sequences from each bin were identified using a BLAST search against the “Pathogenomix Prime 16S” or the novel GTDB-based *rpoB* database, respectively, allowing for implementation of CLSI criteria for valid species-level assignments (supplemented with searches against the relevant NCBI GenBank database if only a low homology score was obtained with the standard databases).

### In-house pipeline – design and bioinformatic processing

The FJORD in-house pipeline was designed specifically for species-level bacterial identification based on ONT amplicon sequencing, including the 16S rRNA gene and *rpoB* amplicons. Briefly, sequencing reads were aligned to a curated bacterial reference database using x-mapper [29], and alignments were sorted and indexed using Samtools [30]. Consensus sequences were generated from mapped reads using samtools consensus, applying minimum depth and mapping quality thresholds. Both an IUPAC consensus sequence (call fraction 0.75) and a majority-rule consensus (call fraction 0.5) were produced. Consensus sequences were quality filtered to remove sequences below a minimum length or containing excessive ambiguous bases (N). High-quality sequences were clustered using VSEARCH [31] based on sequence identity with an alignment-based identity definition. Cluster representative sequences were selected based on minimal ambiguous base content and highest sequencing depth, followed by consolidation of highly similar sequences using an IUPAC-aware identity comparison to avoid artificial cluster splitting. Representative sequences were converted to a majority-rule nucleotide representation and taxonomically classified using BLASTn against the reference database. Final taxonomic assignments were determined based on percent identity thresholds and summarized in a consolidated report including cluster abundance and sequence quality metrics. A detailed description of the in-house pipeline is provided in Supplementary Material 1.

### Statistical analysis

All statistical analyses were based on confirmed detections as defined above, made by the RipSeq software. Calculations were performed in R v4.5.2. The phyloseq package (v1.54.2) was used to calculate alpha-diversity metrics, including the Chao1 and Shannon indexes, and to estimate richness by method. *P* values were adjusted using the Kruskal-Wallis test. Generalized Linear Mixed Models (GLMMs) were used to assess differences in OTU detection probabilities across sequencing methods. The OTU detection table (binary presence/absence; 1/0), derived from an OTU table generated from sequencing data, was used. Models were fitted using the lme4 package (v2.0.1). Moreover, a negative binomial GLMM with library-sized offset was used to assess differences in OTU read counts among sequencing methods. The OTU table (read counts) and the glmmTMB package (v1.1.14) were used. Pairwise comparison between methods was assessed using estimated marginal means (EMMs) with the emmeans package (v2.0.3). A detailed description of the statistical models is provided in Supplementary Material 2. Concordance between methods was assessed based on the number of bacterial detections supplemented by Pearson and Spearman correlation coefficient tests. Confidence intervals for sensitivity and precision were adjusted using the binom package (v1.1-1.1). The ggplot2 package (v4.0.1) was used for data visualization.

## Results

**Fig. 1:**
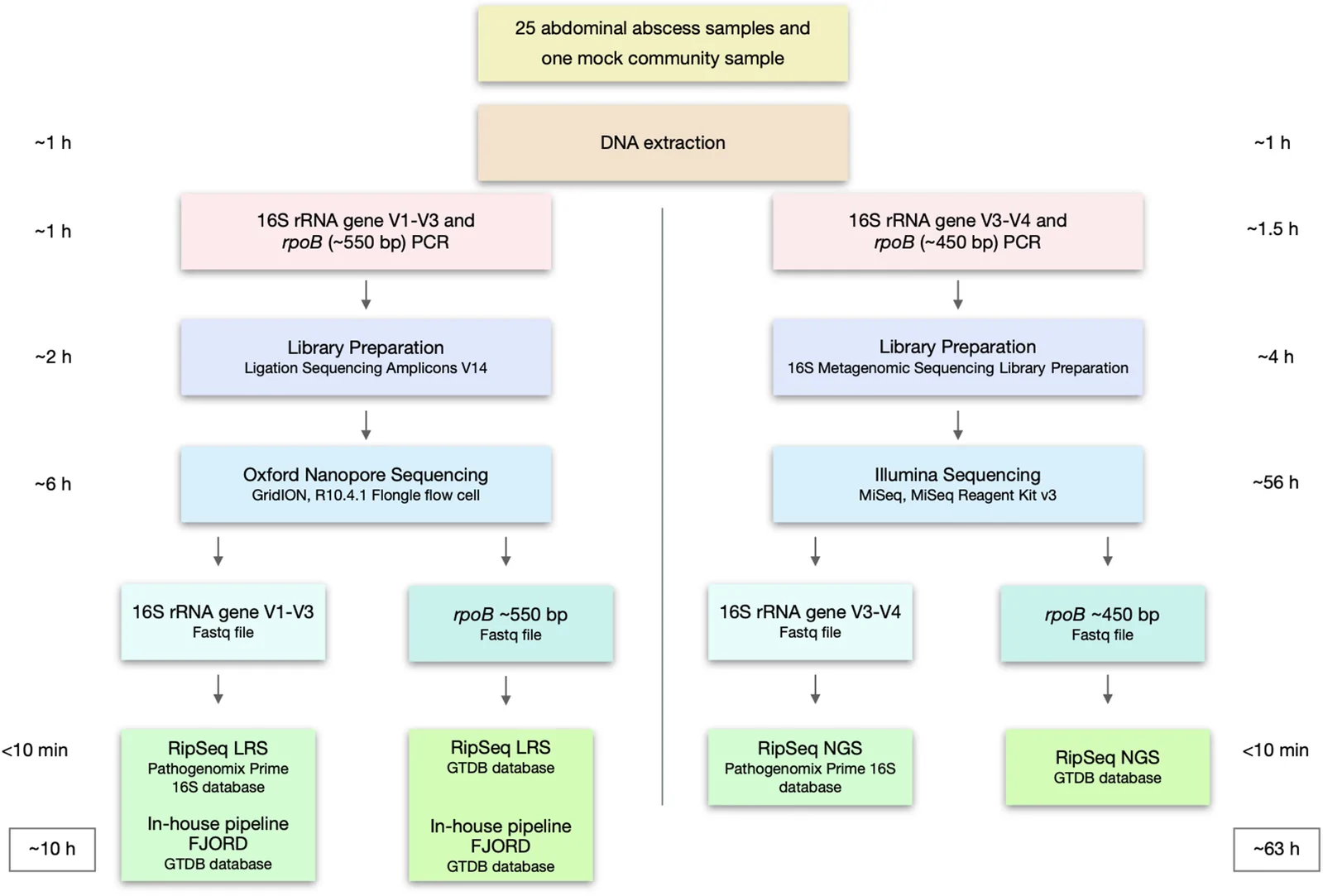
Overview of experimental workflow. Turn-around times for analyses are shown on the sides. Time to results is indicated in the boxes at the bottom of the figure

16S rRNA gene and *rpoB* amplicons from one commercial staggered mock community and 25 intraabdominal abscess samples were successfully sequenced using the ONT and Illumina sequencing platforms (Fig. 1). The average number of reads per sample was 377,859 for ONT and 610,080 for Illumina (Supplementary Table 4, Supplementary Fig. 1).

### Mock community

**Fig. 2:**
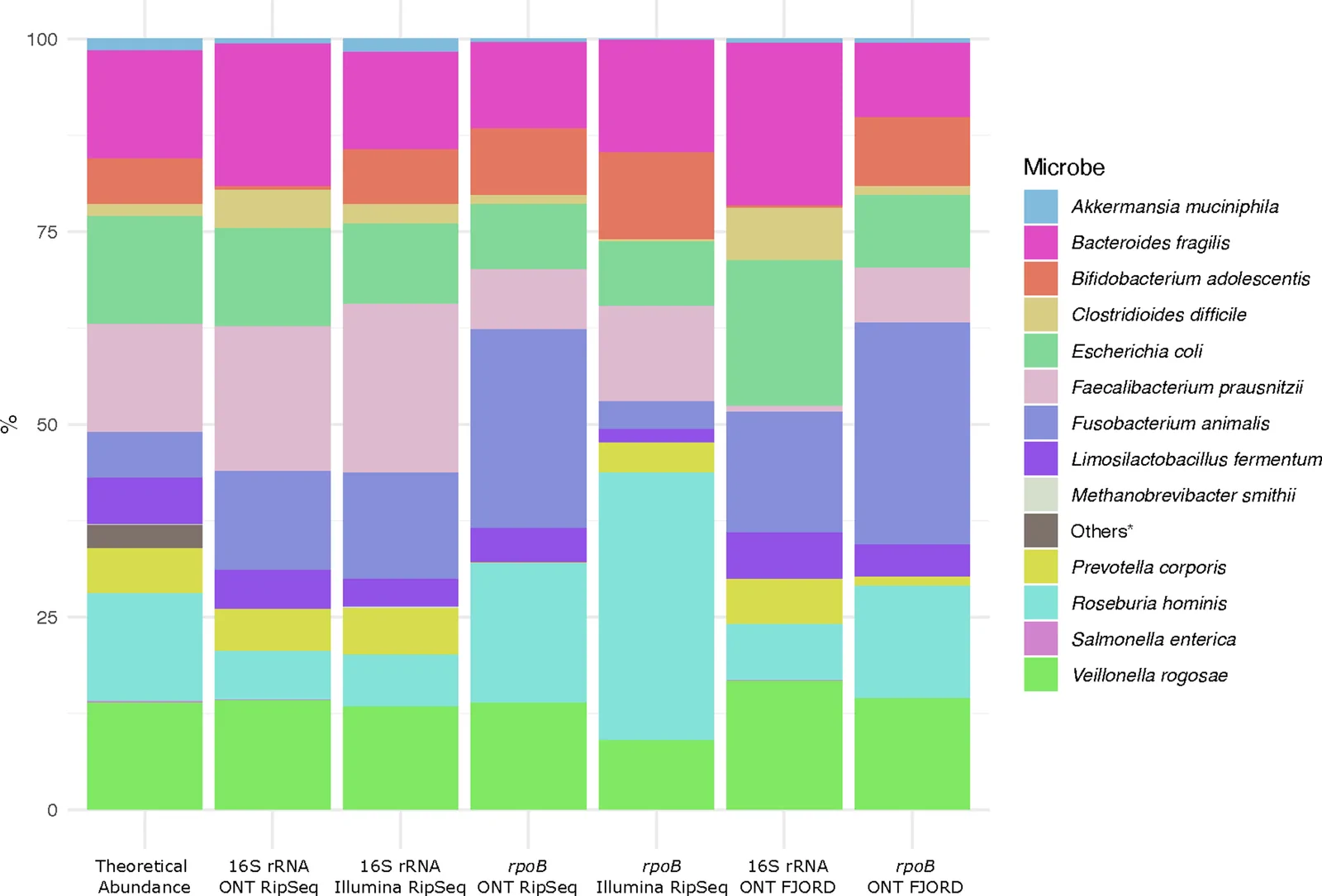
Read distribution (%) for bacteria detected by 16S rRNA ONT RipSeq, 16S rRNA Illumina RipSeq, *rpoB* ONT RipSeq, *rpoB* Illumina RipSeq, 16S rRNA ONT FJORD, *rpoB* ONT FJORD, and theoretical abundance in mock community samples. *Others represent bacterial species comprising less than 0.01% of theoretical abundance and two yeast species

All mock community bacteria representing more than 0.1% of the theoretical abundance were detected by all four approaches. Moreover, *Salmonella enterica*, representing 0.01% of theoretical abundance, was detected using 16S rRNA ONT. The 16S rRNA gene Illumina sequencing approach has been thoroughly evaluated previously [25] and most accurately reflected the composition and theoretical abundance of the mock community sample (Fig. 2). The primers used in this study were designed for prokaryotes, and the archaeon *Methanobrevibacter smithii* was not amplified due to multiple primer mismatches. A lower-than-expected abundance of *Bifidobacterium adolescentis* by 16S rRNA gene ONT sequencing is explained by known forward primer mismatches for *Bifidobacteriales* [21]. The *rpoB* PCR assays were more prone to amplification bias due to a more complex primer design containing multiple degenerate bases, likely leading to interspecies variation in primer-binding efficiency. Additionally, greater variability in amplicon GC-content among species may have further contributed to differential amplification performance.

### Clinical samples

In total, there were 257 confirmed bacterial detections, representing 74 genera and 140 species. Twenty samples were polymicrobial, with diversities spanning from two to 30 species. Among the polymicrobial samples, complete descriptions by all four methods were achieved for only four. In addition, complete descriptions were achieved for eight samples by 16S rRNA ONT, for two samples by 16S rRNA Illumina, for two by *rpoB* ONT, and for three by *rpoB* Illumina (Supplementary Fig. 2). In six samples, no method detected all pathogens; these were complex samples with a number of confirmed detections ranging from 11 to 30.

**Fig. 3:**
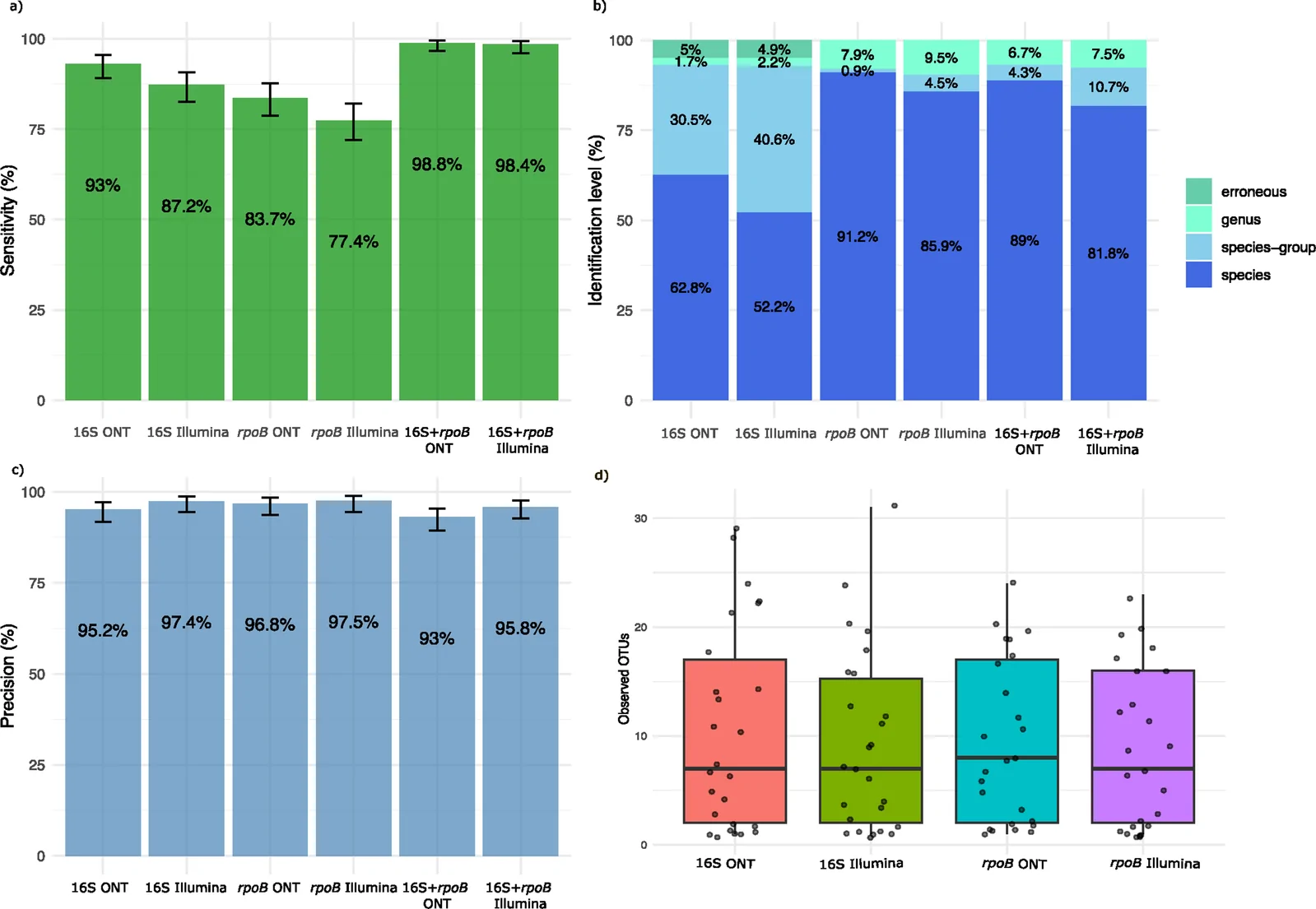
Results from 16S rRNA gene and *rpoB* ONT and Illumina sequencing based on the RipSeq. (**a**) Sensitivity for 16S rRNA ONT, 16S rRNA Illumina, *rpoB* ONT, *rpoB* Illumina, and theoretical estimation of sensitivity using combination approaches: (i) 16S rRNA ONT + *rpoB* ONT and (ii) 16S rRNA Illumina + *rpoB* Illumina. (**b**) Taxonomic assessment of detected bacteria at three classification levels: species, species-group, and genus. Bacteria likely misclassified to species or species-group levels by 16S rRNA gene sequencing are highlighted in turquoise (erroneous). (**c**) Bar plots showing the precision achieved by each sequencing approach. (**d**) Observed richness measured across all four sequencing approaches

16S rRNA ONT sequencing was the most sensitive method and detected 93.0% of the confirmed microbial detections, whereas *rpoB* Illumina was the least sensitive method and detected 77.4% (Fig. 3a). A slightly better precision was obtained for Illumina sequencing (Fig. 3c). *rpoB* amplicons provided the best taxonomic resolution with species-level classification for 91.2% (ONT) and 85.9% (Illumina) of bacteria detected by each method (Fig. 3b).

Combining *rpoB* and 16S rRNA gene sequencing for describing polymicrobial infections may represent an attractive approach for maximizing sensitivity and taxonomic resolution. The estimated detection rates for *rpoB* and 16S rRNA gene combined were 98.8% for ONT and 98.4% for Illumina, with species-level identification achieved for 89.0% and 81.8%, respectively (Fig. 3a-b).

**Fig. 4:**
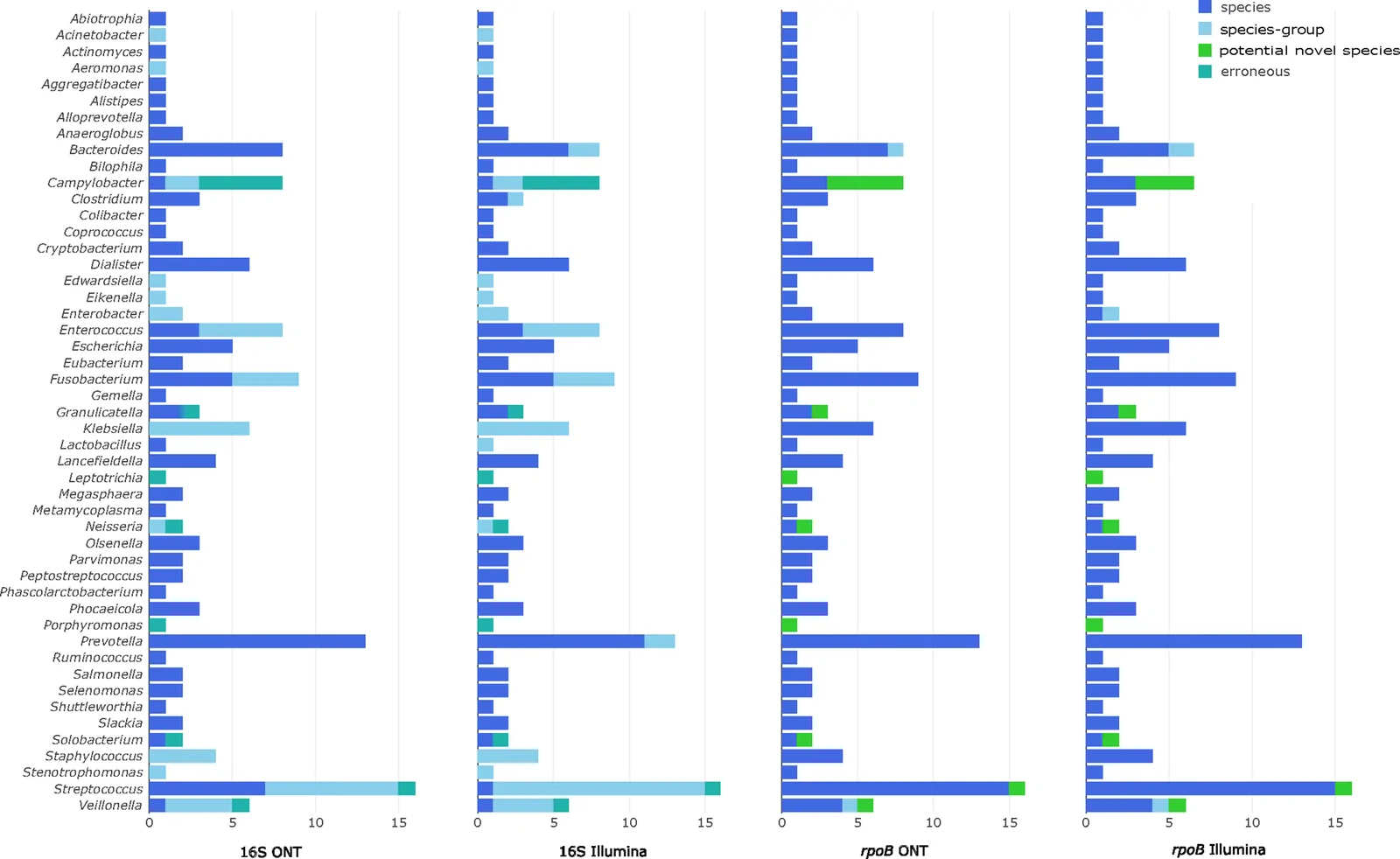
Taxonomic classification for detections made by all four methods across all samples. Numbers below the figure indicate the number of detected bacteria within each genus. Bacteria likely misclassified to species or species-group by 16S rRNA gene sequencing are highlighted in turquoise (erroneous). Bacteria representing potential novel species are highlighted in green

*rpoB* improved resolution within the genera *Acinetobacter*, *Aeromonas*, *Campylobacte*r, *Edwardsiella*, *Eikenella*, *Enterobacter*, *Enterococcus*, *Fusobacterium*, *Klebsiella*, *Staphylococcus*, *Stenotrophomonas*, *Streptococcus*, and *Veillonella*. The shorter *rpoB* Illumina amplicon (∼450 bp) performed similarly to the *rpoB* ONT amplicon, with a slightly lower resolution for *Bacteroides* and *Enterobacter*. The 16S rRNA gene V3-V4 Illumina amplicon provided the lowest resolution and was inferior to the 16S rRNA gene V1-V3 ONT amplicon for multiple genera, most notably *Fusobacterium*, *Streptococcus* and *Bacteroides* (Fig. 4). The poorer resolution of the 16S rRNA gene resulted in the misclassification of several possibly novel species (Supplementary Table 5). The most frequent example was five formally valid identifications of *Campylobacter gracilis* by 16S rRNA gene analysis, where the corresponding *rpoB* sequences obtained only 92.8%-94.6% homology with the *C. gracilis* type strain, pointing to a novel *Campylobacter* species. Detailed sequencing results for each sample are provided in Supplementary Table 3.

### Missed bacterial detections

Some missed detections were explained by significant primer mismatches: the *rpoB* ONT forward primer has a mismatch with *Corynebacterium*, the *rpoB* ONT reverse primer has a mismatch with *Lactobacillus*, and the 16S rRNA gene ONT forward primer has a mismatch with *Bifidobacterium*. Despite perfect primer matching *Actinomyces israelii* and *Schaalia odontolytica* were not detected by *rpoB* sequencing. Presumably, the high *rpoB* GC-content (71.2%, 65.5%, respectively) for these two species resulted in those amplicons being outcompeted in the PCR by *rpoB* amplicons with lower GC-contents from other species in the same samples. However, the majority of missed detections occurred among low-level identifications in complex polymicrobial samples. In broad-range amplification and sequencing, low-abundance bacteria are prone to being outcompeted during PCR or sequencing, and their detection may depend upon variations in primer-binding, amplification efficacy, or sequencing depth, as described previously [25].

### Background contaminants

For ONT sequencing, the most frequently detected contaminant was *Cutibacterium acnes*. However, contaminant patterns were not consistent, with substantial variation observed between samples, indicating that all contaminants were present at low levels and consequently prone to random inclusion under the law of small numbers [25]. Presumed contaminants filtered from the clinical samples and species detected in the negative controls are listed in Supplementary Table 2.

### Concordance between methods

**Fig. 5:**
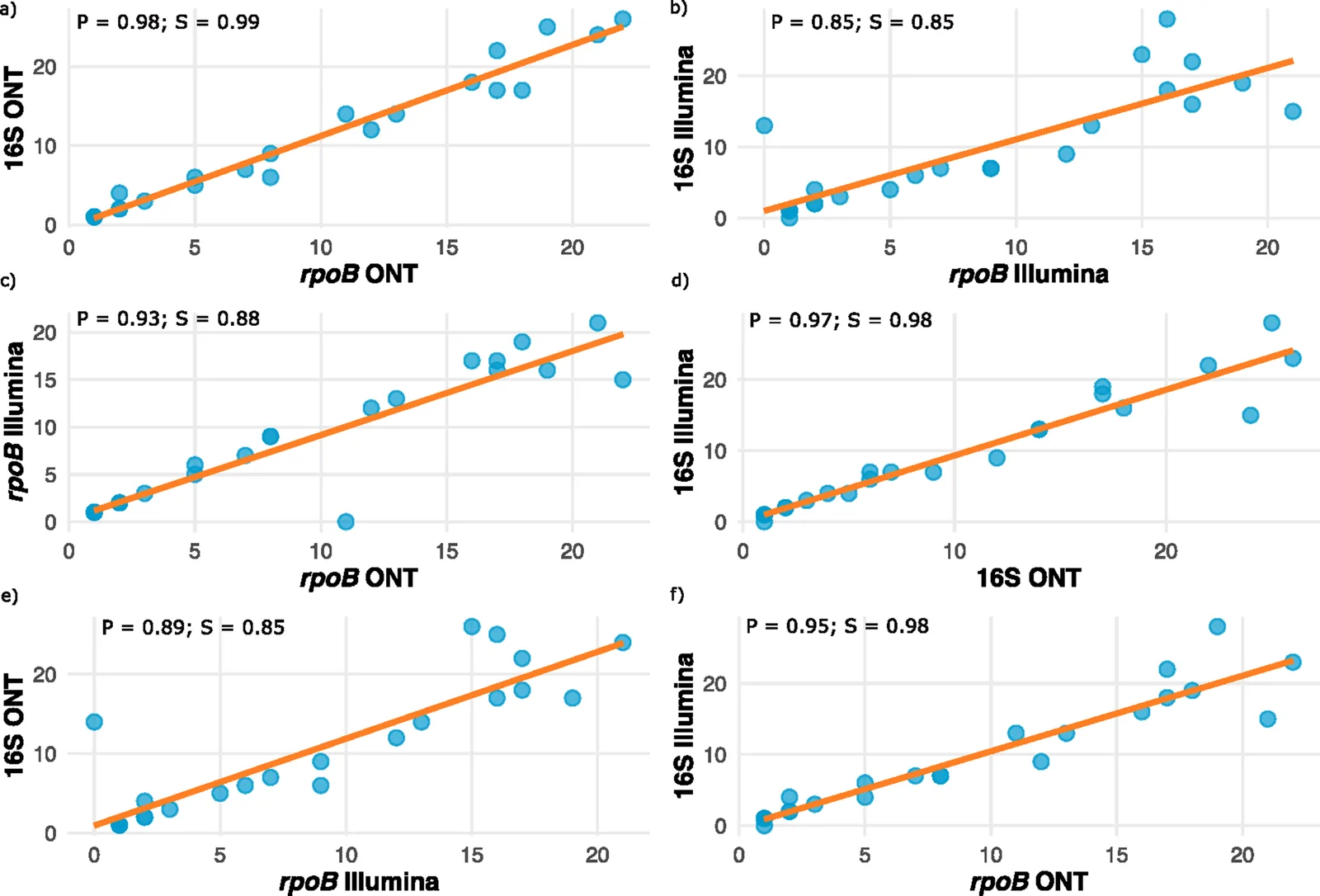
Concordance between methods and correlation coefficients tests (P = Pearson; S = Spearman), based on the number of confirmed detections and results from the RipSeq pipeline. (**a**) Concordance between 16S rRNA ONT and *rpoB* ONT. (**b**) Concordance between 16S rRNA Illumina and *rpoB* Illumina. (**c**) Concordance between *rpoB* Illumina and *rpoB* ONT. (**d**) Concordance between 16S rRNA Illumina and 16S rRNA ONT. (**e**) Concordance between 16S rRNA ONT and *rpoB* Illumina. (**f**) Concordance between 16S rRNA Illumina and *rpoB* ONT

To compare microbial diversities identified by the four sequencing approaches, Shannon and Chao1 alpha-diversity analysis were performed (Supplementary Fig. 3 and Supplementary Fig. 4). The highest richness was obtained by 16S rRNA gene ONT, followed by *rpoB* ONT (Fig. 3d). However, there were no significant differences between methods (Kruskal-Wallis, *p* = 0.86). Concordance between methods, as presented in Fig. 5a-f, shows consistently strong positive correlation in richness between sequencing methods (Pearson: 0.85–0.98; Spearman: 0.85–0.99) (Fig. 5). A very strong agreement was observed for 16S rRNA gene ONT and *rpoB* ONT sequencing, followed by 16S rRNA gene ONT and 16S rRNA gene Illumina sequencing. Some biases were observed between gene targets, which may contribute more to variability than the sequencing platforms.

A binomial GLMM revealed no significant effect of sequencing method on OTU detection probability. A negative binomial GLMM with offset for sequencing depth showed no significant effect of sequencing method on OTU relative abundance. GLMM pairwise comparisons identified no significant differences between methods in OTU detection or abundance. All results are presented in Supplementary Material 2.

### Evaluation of the in-house bioinformatics pipeline “FJORD”

All mock community bacteria representing more than 0.1% of theoretical abundance were detected using the in-house pipeline for both genes. Additionally, based on the 16S rRNA ONT data, the in-house pipeline also correctly reported *Salmonella enterica* present at 0.01%. The relative abundances of bacteria reported by the in-house pipeline were generally consistent with the results obtained using RipSeq, except for *Faecalibacterium prausnitzii*, which was reported by a very low abundance of only 0.8% based on the 16S rRNA ONT data (Fig. 2). The majority of *F. prausnitzii* reads were lost in the binning and consensus sequence generation step due to priorities in the noise versus signal filtering. In the clinical samples, the in-house pipeline detected 86.8% (223) of confirmed bacterial detections based on the 16S rRNA ONT data and 83.7% (215) based on *rpoB* ONT data. Overall, the in-house pipeline demonstrated high concordance with the results obtained by the RipSeq pipeline. The in-house pipeline assigned a smaller proportion of the reads, and most undetected bacteria were low-abundance species in polymicrobial samples. Detailed results for the in-house pipeline are presented in Supplementary Table 3.

### Detection and identification of species not represented in the binning databases

By design, both the RipSeq and the FJORD programs allowed for the detection of species missing in the binning databases by assigning them to a lower classification rank or categorizing them as unclassified. Many of these could later be correctly assigned to the species-level using BLAST searches against other/larger reference databases, as shown in Supplementary Table 6. For the 16S data, this occurred less frequently with the RipSeq software that used a more comprehensive 16S rRNA gene reference database. For *rpoB*, where both programs used the same databases, the frequency of such detections were similar.

## Discussion

Here, we present and evaluate a complete approach for ONT sequencing of the 16S rRNA and *rpoB* genes directly from patient samples in clinical microbiology.

Total preparation time from DNA extraction to the start of ONT sequencing was 4–5 h. Although this will increase somewhat with the number of samples, sequencing can still be started the same working day, providing overnight results. In this study, each sample and gene was sequenced individually for six hours on a separate Flongle flow cell. However, when running overnight, the Flongle output should be sufficient for sequencing both gene targets for up to three samples on the same cell using barcoding. For laboratories with larger sample volumes, a more cost- and time-efficient approach would be to sequence all samples and both targets simultaneously on a larger MinION flow cell.

For bacterial identification based on Sanger sequencing of the 16S rRNA gene, the CLSI recommends using the 500 bp V1-V3 segment, considered the most variable part of the gene [19]. For species-level discrimination, full-length 16S rRNA sequencing enabled by ONT has generally been considered an improvement [6, 8, 13, 14]. However, for alignment-based approaches applying CLSI interpretive criteria, it is not intuitive that full-length 16S rRNA sequencing will improve species-level discrimination, since including lower-variability regions may reduce the percentage difference to the next alternative species [32]. This might be different for Bayesian classifiers, theoretically able to exploit any statistically significant difference between neighbouring species. A study using such a classifier suggests that full-length 16S rRNA gene sequencing provides species-level resolution and outperforms partial 16S rRNA analysis [33]. However, this in silico analysis was limited to a preselected subset of 16S rRNA gene sequences that were all ≥ 1% different from each other over the full-length gene, assuring sufficient discrimination. The study design further biased comparisons with alternative partial segments since references differing by more than 1% in a subsegment were excluded if there was less than 1% difference across the entire gene. Even in this comparison, the V1-V3 segment achieved 90% species-level resolution, and the authors suggested that this segment may be sufficient for species-level discrimination. In a later study, the resolution provided by the V1-V3 region was found to be comparable with that of the full-length gene [13]. Longer amplicons generally reduce PCR amplification efficacy [34]. Additionally, primers for amplification of the full-length 16S rRNA gene may be less universal than well-established alternatives for partial amplification [19, 35, 36]. In clinical microbiology, with samples often containing low levels of bacterial DNA and abundant human DNA, PCR sensitivity and specificity should be prioritized over a questionable gain in species-level resolution. Therefore, here, a well-established real-time 16S rRNA gene V1-V3 PCR was used for ONT sequencing. The use of this might simplify the transition to ONT sequencing for clinical laboratories that have a validated real-time 16S rRNA gene V1-V3 PCR in place. To increase species-level resolution, supplementing partial amplification of the 16S rRNA gene with partial *rpoB* amplification is helpful. In such an approach, the 16S rRNA gene amplification ensures high sensitivity, while the *rpoB* target improves species discrimination. As demonstrated by the findings in Supplementary Table 5, this approach also allows for recognition of novel species, obtaining a low score in the BLAST search. In addition, combining two targets reduces the risk of missing species due to primer mismatches and increases detection rates for low-concentration species in complex polymicrobial infections. Replacing the 16S rRNA gene with *rpoB* is not advisable, as the *rpoB* PCR is less efficient and shows more cross-reactivity with human DNA, leading to a lower sensitivity for weakly positive samples [18].

Previous studies have highlighted performance differences among pipelines for analysis of the ONT 16S rRNA amplicon data and demonstrated challenges in detecting present species even in monomicrobial samples [37]. In the present study, we used three different bioinformatics approaches: The NGS module of the RipSeq software, optimized for analysis of Illumina/IonTorrent amplicon data [1, 4, 22], the new RipSeq LRS module optimized for analysis of ONT amplicon data, and the new in-house FJORD pipeline optimized for ONT amplicon data. The RipSeq software for ONT amplicon data analysis is based on a two-step principle, with binning against a database as a first step, followed by the generation of a consensus sequence from each bin, finally identified using BLAST. Such an approach mitigates some problems related to the analysis of ONT amplicon data, as random sequencing errors are nullified through the consensus generation from each bin. Furthermore, when a minor proportion of reads from a given species are erroneously binned to a reference representing another related species due to the ONT-associated errors and/or small 16S rRNA gene inter-species variations and/or failures in the k-mer based binning, the consensus sequence will still represent the biological sequence and be correctly re-assigned in the subsequent BLAST search. RipSeq is a Windows/MacOS-based software and does not require specialized bioinformatics expertise, facilitating its implementation in routine laboratory settings. However, commercial pipelines are associated with higher analysis costs and may impose restrictions on data storage and the handling of sensitive data. These aspects should therefore be considered when selecting an appropriate bioinformatics solution. As an alternative solution, we designed and evaluated an in-house pipeline based on similar principles. The in-house pipeline showed high concordance for the *rpoB* gene and yielded comparable results for the 16S rRNA gene. Some differences for 16S rRNA gene may be explained by the use of different reference databases for the binning step or by the different mapping algorithms used by the two bioinformatics tools. Importantly, both bioinformatics approaches enabled the detection of species not represented in the binning databases.

Metagenomic sequencing is an alternative approach that, given sufficient sequencing depth, will provide unambiguous species-level identifications and can also allow for identification of genes or mutations associated with antimicrobial resistance. Several studies have demonstrated promising results using this method [38, 39]. However, since there is no selective sequencing of a specific target, massive sequencing capacity is necessary to ensure detection of all organisms present in complex polymicrobial infections. The lack of sensitive methods for selective purification of bacterial DNA further increases the number of sequencing reads needed per sample, making each analysis much more costly and reducing sensitivity in samples with high amounts of human DNA and low amounts of bacterial DNA. Another approach is long-read sequencing of the ribosomal RNA operon (RRN), comprising the full-length 16S rRNA gene, the internal transcribed spacer (ITS), and varying parts of the 23 S rRNA gene, using amplicons ranging from 2500 to 4500 bp. Previous investigations have shown that RRN can improve phylogenetic resolution and enable more accurate species-level identification [40, 41]. Despite its potential, this is a less-investigated approach, and its utility in diagnostic microbiology has not been evaluated. The longer amplicons will be challenging with regard to PCR sensitivity, and no studies have critically assessed primer universality, nor primer cross-reactivity with human DNA.

A strength of this study is the multifaceted evaluation of the ONT results. All samples were analysed using two separate gene targets, two different sequencing technologies, and three different bioinformatic approaches, showing highly overlapping results. We used a two-step bioinformatic approach for effective de-noising of ONT sequencing data, to obtain BLAST-based identifications allowing for implementation of CLSI interpretive criteria, and to allow for detection of species not represented in the databases. Consensus sequence generation also allows for capture of sequence variants of marker genes that are missing from the reference database. We also provide an in-house pipeline built on a similar two-step principle as an alternative to the commercial RipSeq software used as the primary analysis tool in this study.

This study also has several limitations. Except from the commercial mock community, we cannot know the true composition of the samples. There is no gold-standard method for bacterial detection in clinical samples, and although amplicon sequencing is a reference method in bacteriology, low-abundance species in polymicrobial samples can remain undetected for various reasons [25]. The use of a composite reference standard is therefore likely to have resulted in the exclusion of some relevant findings detected by only one method. Furthermore, multiplexing samples on the same MinION flow cell as suggested in the discussion may introduce sample cross-contamination and bleeding, which were not assessed here. Preliminary data generated by the authors (not shown) suggest that this is not a substantial challenge. Finally, the *rpoB* PCR used with the *rpoB* Illumina assay was only rudimentarily evaluated in vitro. It was found to have an amplification efficacy similar to the *rpoB* PCR used with the *rpoB* ONT assay, but with a higher tendency for cross-reactivity with human DNA, likely to impair sensitivity in some clinical samples with a high human to bacterial ratio.

## Conclusions

ONT sequencing of partial 16S rRNA gene and *rpoB* amplicons directly from clinical samples is feasible, with results paralleling those of Illumina sequencing of the same phylogenetic markers. The 16S rRNA ONT sequencing provided the best sensitivity and improved species-level resolution compared to 16S rRNA Illumina sequencing. Including *rpoB* as a supplementary target enhanced both sensitivity and species-level discrimination for the two platforms. ONT sequencing supports the short turn-around time and flexibility essential in clinical microbiology and allow for cost-efficient, small and frequent runs.

## Supplementary Information

Below is the link to the electronic supplementary material.

Supplementary Material 1

Supplementary Material 2

Supplementary Material 3

Supplementary Material 4

Supplementary Material 5

Supplementary Material 6

Supplementary Material 7

Supplementary Material 8

Supplementary Material 9

Supplementary Material 10

Supplementary Material 11

Supplementary Material 12

## Data Availability

The datasets generated and analysed during the current study are available in the European Nucleotide Archive (ENA) repository at European Molecular Biology Laboratory-European Bioinformatics Institute (EMBL-EBI) under accession number PRJEB104016. The in-house pipeline FJORD is available at GitHub depository https:/github.com/adnsvrtsn/fjord.

## Abbreviations

ANI: Average Nucleotide Identity
BLB: Bacterial Lysis Buffer
bp: base pair
FJORD: Flexible Joint Operational pipeline for Reference-based Diagnostics
GTDB: The Genome Taxonomy Database
ITS: the internal transcribed spacer
ONT: Oxford Nanopore Technologies
OTUs: Operational Taxonomic Units
RRN: the ribosomal RNA operon
rRNA: The 16S ribosomal RNA gene
16S TNGS: 16S rRNA targeted next-generation sequencing
