# Supplementary material for: Bacterial identification in clinical samples – comparing Nanopore and Illumina sequencing of the 16S rRNA and rpoB genes
Source: BMC Infect Dis. 2026 Jul 28;26:1877. doi: 10.1186/s12879-026-14101-z (PMC13700529; doi:10.1186/s12879-026-14101-z)
Supplement: Supplementary file 2 [file 12879_2026_14101_MOESM11_ESM.pdf]

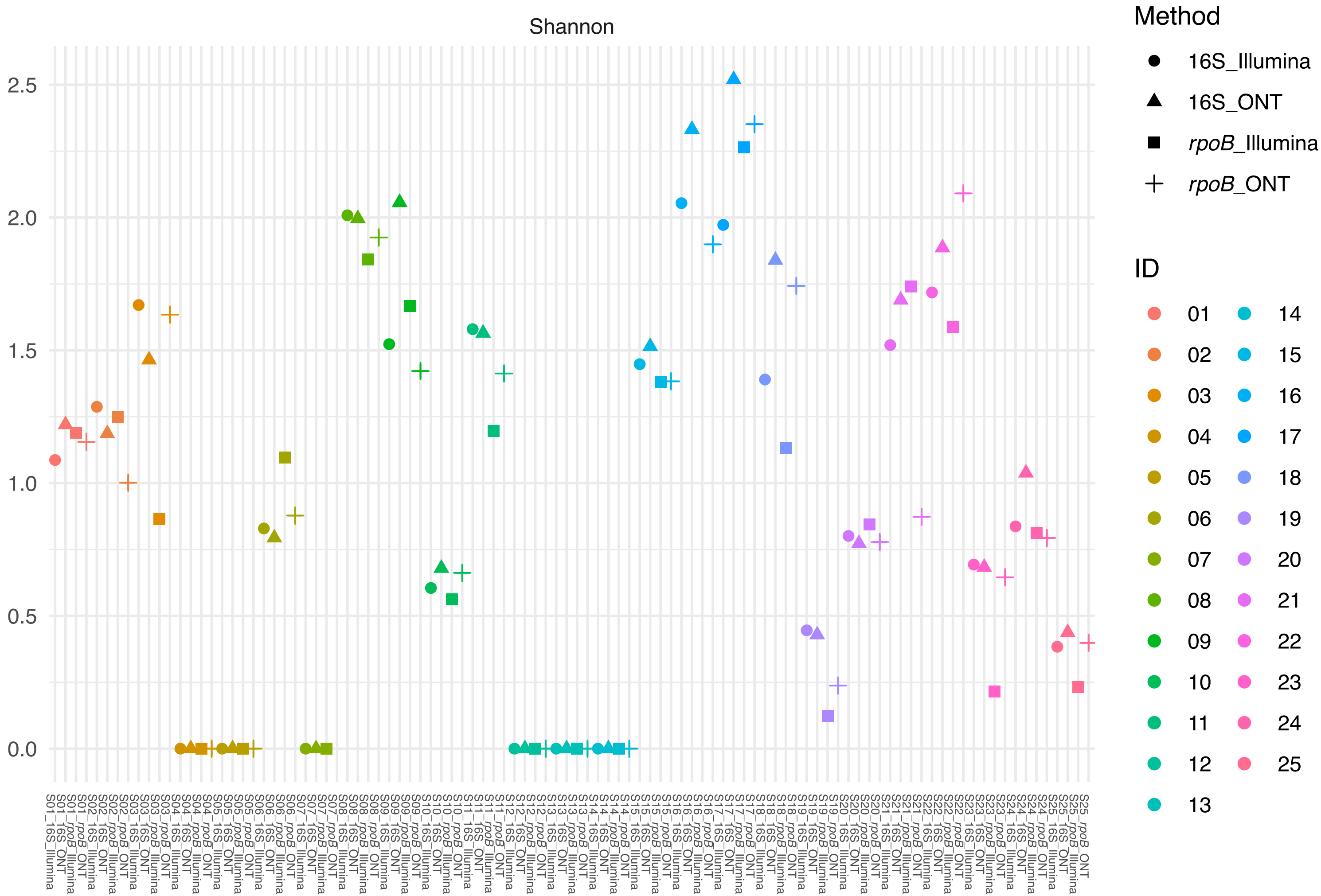

Supplementary Fig. 3. Alfa-diversity metric Shannon index for all samples included in the study
