# Supplementary material for: Bacterial identification in clinical samples – comparing Nanopore and Illumina sequencing of the 16S rRNA and rpoB genes
Source: BMC Infect Dis. 2026 Jul 28;26:1877. doi: 10.1186/s12879-026-14101-z (PMC13700529; doi:10.1186/s12879-026-14101-z)
Supplement: Supplementary file 5 [file 12879_2026_14101_MOESM3_ESM.docx]

| **Primer name** | **Sequence** |
| --- | --- |
| **RpoB_DPO1-F**  *Desulfovibrio* spp.  *Bilophila wadsworthia*  *Corynebacterium* spp.  *Corynebacterium otitidis*  *Metamycoplasma* spp.  *Mycoplasmoides* spp. | 5´−TCNCARTTYATGGAYCAIIHIAAYCC−3´  5´−−−−−−−−−−−−−−−−−−IIHI−−−T−−3´  5´−−−−−−−−−−−−−−−−−−IIHI−−−T−−3´  5´−−−−−−−−−−−−−−−−−−IIHI−−−T−−3´  5´−−−−−−−−−−−−−−−−−−IIHI−−−G−−3´  5´−−−−−−−−−−−−−−−−−−IIGI−−−−−−3´  5´−A−−−−−−−−T−A−−−−−IIHI−−−−−−3´ |
| **RpoB_DPO2-F** | 5´−AGNCARTTYATGGAYCAIIHIAAYCC−3´ |
| **RpoB_DPO-R**  *Lactobacillus gasseri/johnsonii/paragasseri*  *Saccharibacteria* (TM7) spp.  **RpoB_Illumina_DPO-F**  *Mycoplasmoides* spp.  *Saccharibacteria* (TM7) spp.  *Ureaplasma* spp. | 5´−CNGCYTGDCKYTKCATRTTIIIICCCAT−3´  3´−−−−−−−−−−−−−−GTG−−−IIII−−−−−−5´  3´−−−−−−−−−−−−−−−−−−−−IIII−−AG−−5´  5´-TGYCCIRTHGARWCICCIGARGGICIIAAYAT-3´  5´-------------------------A--------3´  5´----T----G---------------G-----G--3´  5´-------------------------A--------3´ |

**Supplementary Table 1**. List of species and genera with significant mismatches in the *rpoB* primer-binding area.
