# Supplementary material for: Bacterial identification in clinical samples – comparing Nanopore and Illumina sequencing of the 16S rRNA and rpoB genes
Source: BMC Infect Dis. 2026 Jul 28;26:1877. doi: 10.1186/s12879-026-14101-z (PMC13700529; doi:10.1186/s12879-026-14101-z)
Supplement: Supplementary file 6 [file 12879_2026_14101_MOESM4_ESM.docx]

| Number of reads | 16S rRNA gene ONT | 16S rRNA gene Illumina | *rpoB* ONT | *rpoB* Illumina |
| --- | --- | --- | --- | --- |
| Total (average) | 399983 | 561995 | 355734 | 658165 |
| Total min | 37682 | 201031 | 80799 | 49471 |
| Total max | 650026 | 1002233 | 605686 | 1162056 |
| Used (average)  RipSeq | 295733 | 267354 | 230956 | 374640 |
| Used min | 24643 | 49453 | 1726 | 289 |
| Used max | 539022 | 461946 | 510628 | 593594 |
| % used (average) | 74% | 48 % | 65 % | 57 % |
| Used (average)  in-house pipeline | 250429 | NA | 221694 | NA |
| Used min | 6087 | NA | 1321 | NA |
| Used max | 649856 | NA | 535027 | NA |
| % used (average) | 63% | NA | 62% | NA |

**Supplementary Table 4.** Average, minimum and maximum number of reads obtained for the 16S rRNA gene and *rpoB* using ONT and Illumina sequencing. The reported number of used reads represents reads after removal of chimera and invalid reads.
