# Supplementary material for: Bacterial identification in clinical samples – comparing Nanopore and Illumina sequencing of the 16S rRNA and rpoB genes
Source: BMC Infect Dis. 2026 Jul 28;26:1877. doi: 10.1186/s12879-026-14101-z (PMC13700529; doi:10.1186/s12879-026-14101-z)
Supplement: Supplementary file 7 [file 12879_2026_14101_MOESM5_ESM.docx]

**Supplementary Material 2**

**Statistics**

1. **Generalized linear mixed model GLMM**

The response variable was OTU detection (binary presence/absence; 1/0), derived from an OTU table generated from sequencing data. Sample identity and OTU identity were included as random intercepts to account for repeated measurements across samples and non-independence among OTUs, respectively. Models were fitted in R using the lme4 package (v2.0.1).

**Model specification:**

Family: binomial (logit)

Formula: Detect ~ Method + (1 | Sample) + (1 | otu)

Data: df_long

1.1. Pairwise differences between sequencing methods were assessed using estimated marginal means (EMMs) with Tukey-adjusted multiple comparisons implemented in the emmeans package (v2.0.3).

emmeans (model_detect, pairwise ~ Method, type = "response")

2. **Negative binomial GLMM with library-size offset.**

Differences in sequencing depth among samples were accounted for by including the log-transformed total read count per sample as an offset term in the model, effectively modeling relative abundance. Models were fitted in R using glmmTMB package (v1.1.14).

**Model specification:**

Family: nbinom2 (log)

Formula: ReadCount ~ Method + offset(log(TotalReads)) + (1 | Sample) +	(1 | otu)

Data: df_long

2.1. Pairwise differences among sequencing methods were evaluated using estimated marginal means (EMMs) with Tukey-adjusted multiple comparisons implemented in the emmeans package. Results were back-transformed to the response scale and interpreted as relative OTU abundance differences among methods.

3. **Results**
   3.1. GLMM – estimated detection probabilities

| **Method** | **probabilities** | **SE** | **df** | **asymp.LCL** | **asymp.UCL** |
| --- | --- | --- | --- | --- | --- |
| **16S Illumina** | 0.0322163873951127 | 0.00817714063964636 | Inf | 0.0195206182862823 | 0.0527251763611184 |
| **16S ONT** | 0.0348704574839846 | 0.00880492191187763 | Inf | 0.0211776976269321 | 0.0569018117761479 |
| ***rpoB* Illumina** | 0.0283220481278702 | 0.00724890880953787 | Inf | 0.0170962833858258 | 0.046569638130949 |
| ***rpoB* ONT** | 0.0302562220342687 | 0.00771098498440736 | Inf | 0.0182992984987016 | 0.0496309228224304 |

3.2 GLMM pairwise results.

| **contrast** | **odds.ratio** | **SE** | **df** | **null** | **z.ratio** | **p.value** |
| --- | --- | --- | --- | --- | --- | --- |
| 16S Illumina / 16S ONT | 0.92135403 | 0.09272932 | inf | 1 | -0.8138629 | 0.84795828 |
| 16S Illumina / r*poB* Illumina | 1.14207932 | 0.11970504 | inf | 1 | 1.26749789 | 0.5837124 |
| 16S Illumina / *rpoB* ONT | 1.06694216 | 0.11033652 | inf | 1 | 0.62657769 | 0.92356324 |
| 16S ONT / *rpoB* Illumina | 1.23956621 | 0.12810932 | inf | 1 | 2.07799936 | 0.16028012 |
| 16S ONT / *rpoB* ONT | 1.15801541 | 0.11803506 | inf | 1 | 1.43931605 | 0.47470072 |
| *rpoB* Illumina / *rpoB* ONT | 0.93421021 | 0.09906602 | inf | 1 | -0.6417594 | 0.91844325 |

3.3 NB GLMM offset results.

| **contrast** | **ratio** | **SE** | **df** | **null** | **z.ratio** | **p.value** |
| --- | --- | --- | --- | --- | --- | --- |
| 16S Illumina / 16S ONT | 0.77360971 | 0.22853931 | inf | 1 | -0.8688928 | 0.8209448 |
| 16S Illumina/ *rpoB* Illumina | 1.04877251 | 0.33716722 | inf | 1 | 0.14812535 | 0.99884223 |
| 16S Illumina / *rpoB* ONT | 0.82069588 | 0.26729431 | inf | 1 | -0.6067159 | 0.92998346 |
| 16S ONT / *rpoB* Illumina | 1.35568685 | 0.43272795 | inf | 1 | 0.95336264 | 0.77588396 |
| 16S ONT / *rpoB* ONT | 1.06086553 | 0.34266019 | inf | 1 | 0.18292571 | 0.99782886 |
| *rpoB* Illumina / *rpoB* ONT | 0.78252993 | 0.25567126 | inf | 1 | -0.7505514 | 0.87646652 |
