# Supplementary material for: Bacterial identification in clinical samples – comparing Nanopore and Illumina sequencing of the 16S rRNA and rpoB genes
Source: BMC Infect Dis. 2026 Jul 28;26:1877. doi: 10.1186/s12879-026-14101-z (PMC13700529; doi:10.1186/s12879-026-14101-z)
Supplement: Supplementary file 8 [file 12879_2026_14101_MOESM8_ESM.docx]

**Supplementary material 1**

**The *FJORD* pipeline**

# **Motivation**

Several tools exist for taxonomic classification of ONT amplicon sequencing reads, including Emu, NanoClust, EPI2ME, Kraken2/KrakenUniq (and NameCo) [1, 2, 3]. However, for clinical diagnostics, each has shortcomings that limit their utility. First, these tools assume that all bacteria in a sample will closely match sequences in the reference database and will therefore mask or misclassify sequences originating from species lacking representative database entries. Second, a few tools output consensus sequences as a secondary output to verify the taxonomic classification. Third, primer choice affects what is reportable from the wet lab; poorly amplifiable sequences due to primer mismatches are less likely to be detected, as they are outcompeted by other amplicons. An a priori evaluation of which clinically relevant taxa are likely to be missed due to primer mismatch was important to us. Fourth, during consensus sequence generation, we found that primer trimming using tools such as Chopper [4], while largely effective, was not perfect. Even very low levels of residual primer sequence will introduce errors in consensus sequences. In addition, resolution of chimeric and tandem reads is also largely effective but imperfect, providing yet another source of error.

We therefore devised a novel yet simple approach that takes all the above considerations into account. The pipeline needed to be flexible in terms of reference database, as we use both 16S rRNA and marker genes such as *rpoB* in our clinical practice.

# **Database generation**

In our laboratory, we currently use two PCR targets sequenced with ONT for taxonomic classification: 16S rRNA and *rpoB*. To preserve taxonomic nomenclature and clustering, we used the marker gene database of GTDB release 10 (R10-RS226) to retrieve reference sequences [5]. We downloaded the complete database and retrieved the file TIGR02013.fna, which contains full-length *rpoB* sequences. GTDB also offers 16S rRNA/SSU databases with both representative sequences and multiple sequences per species; however, there are some issues with this particular database.

First, some sequences are associated with erroneous genera, which creates misinterpretation if not accounted for. Separate efforts to correct for this exist with the SATIVA-corrected database [6] in which sequences are filtered by length and corrected such that their phylogenetic signal is consistent with their taxonomy or removed if overtly incongruent. We therefore used representative sequences as the starting point for our database (file sbdi-gtdb-sativa.r10rs226-2.1genome.addSpecies.fna.gz, available at https://figshare.scilifelab.se/ndownloader/files/59220341).

Second, the 16S rRNA gene appears in multiple copies in bacterial genomes, and in metagenome-assembled genomes (MAGs) from short-read datasets, these sequences are often on separate contigs disjointed from the rest of the genome. Consequently, not all GTDB genome entries have 16S rRNA sequences attached to them. No corrective action was possible for this second issue.

As many sequence analysis tools do not tolerate whitespace in FASTA headers, the headers were modified to replace whitespace with “_”, and only accession number and taxonomic information was retained. If the GTDB metadata file is downloaded as well, the FASTA headers may be altered with awk scripts to create compatible headers for Kraken2-database generation, which could be used to follow the ONT sequencing run in real time. Scripts to modify GTDB or Sativa multifasta headers in this fashion are available in the FJORD GitHub repo.

To create the reference databases used by FJORD (which expects a multifasta file with modified GTDB-formatted headers), we downloaded 64-bit USEARCH v11.0.667 [7] from https://github.com/rcedgar/usearch_old_binaries/ and used the -search_pcr function to perform in silico PCR. This function is one of few tools that support IUPAC-degenerate bases in primer sequences, which was necessary given our DPO primer design. It uses a separate FASTA file containing primer sequences to extract amplicon-length products. While Seqkit [8] can also perform in silico PCR, it does not output a detailed table with PCR hit information including 3' mismatch positions, which is valuable for evaluating primer performance across taxa.

In our clinic, we use the following DPO primers (the poly-I linker section in each primer is replaced by N's):

>*rpoB*_fwd_combo_l26

WSNCARTTYATGGAYCANNHNAAYCC

>*rpoB*_rev_l28

CNGCYTGDCKYTKCATRTTNNNNCCCAT

>16S_v1-v3-F_l30

AGAGTTTGATCMTGGCTCANNNNNAACGCT

>16S-v1-v3-R_l22

CGGCTGCTGGCANNNANTTRGC

We used the standard setting of a maximum of 2 mismatches (-maxdiffs flag), as allowing more mismatches in these already degenerate primers could in some instances generate multiple hits per database sequence, which downstream applications would not tolerate. When the -pcrout flag is enabled, a table containing all PCR products is generated. This table is useful for sorting hits by the number of mismatches in the 3' end of each primer, as such mismatches are more likely to cause amplification failure.

We then used -fastx_truncate to remove the primer sequences from the extracted amplicons. It is possible to extend primer sequence trimming further into the product itself, as it is known that several nucleotides are simultaneously within the pore of the ONT device during sequencing, and DPO motifs within the primers may cause lower-quality basecalling of adjacent amplicon positions.

The resulting multifasta files containing primer-trimmed amplicon sequences were then used as reference databases for the FJORD pipeline.

# **Taxonomic classification and consensus sequence generation**

In ONT-based amplicon analysis, minimap2 [9] is the standard tool used for read alignment to reference databases. Other tools, such as Emu, also rely on minimap2 for this purpose. We reasoned that by mapping reads to a primer-trimmed reference database, we could generate consensus sequences directly using “samtools consensus” [10], and thereafter cluster consensus sequences based on their sequence identity.

While minimap2 often gave adequate results, we frequently observed large taxonomic expansions in clinical samples where the reference database contained sequences within ~98--99% identity of each other. This problem is particularly common with the 16S rRNA reference database. We also frequently observed many short sequences arising from spurious secondary alignments. When we re-analysed those same samples with alternative methods (such as *rpoB* amplicon sequencing), the numerous similar taxa would collapse into one or two species, which likely represented the true positive taxa present in the sample. Emu would similarly report many taxa, of which many were likely false positives. Taxonomic groups where this problem occurs include, but are not limited to, *Enterobacteriaceae*, *Pseudomonas,* the *Streptococcus mitis* group, *Fusobacterium*, *Enterococcus faecium/lactis*, *Haemophilus*, and *Neisseria*.

In an attempt to solve this, we employed X-mapper [11], which uses multiple K-mer array sizes and gapped K-mers as seeds for primary alignment, as opposed to minimap2 which uses a single K-mer size. Although X-mapper was explicitly designed for Illumina data, short (<700-800 bp) amplicon sequences basecalled with SUP accuracy on R10.4 flow cells worked well with this tool.

## **Pipeline workflow**

After alignment of reads to the primer-trimmed reference database with X-mapper, FJORD generates dual consensus sequences using “samtools consensus”. The first set uses IUPAC ambiguity codes at positions where less than 75% of reads agree on a base, which preserves information about mixed positions for downstream clustering. The second set uses majority-rule calling, which always reports the most common base and is used for BLAST-based taxonomy assignment since BLAST does not handle IUPAC codes. Both consensus sets require a minimum read depth of 10 and a minimum mapping quality of 25.

Consensus sequences are then quality-filtered using embedded Biopython modules [12], removing sequences shorter than 400 bp or containing more than 2% ambiguous bases (N's). This strict N% filter serves as a proxy for mapping correctness: sequences that genuinely belong to the reference target produce clean consensus regardless of their evolutionary distance, whereas mis-mapped reads produce N-rich sequences due to positional conflicts. This filter thus removes mapping artefacts while retaining genuine novel species.

The filtered IUPAC-bearing consensus sequences are clustered using VSEARCH [13] with gapped alignment (--iddef 2) at 99% identity, which correctly handles the homopolymer indel errors characteristic of ONT sequencing. After initial clustering, an IUPAC-aware consolidation step merges any clusters whose representative sequences are >99% identical when ambiguity codes are treated as compatible with any matching base. This prevents artificial splitting of clusters due to stochastic variation in IUPAC calls between consensus sequences derived from the same underlying taxon.

Representative sequences for each cluster are selected based on the lowest percentage of ambiguous bases, with alignment depth as a tiebreaker. The majority-rule versions of these representatives are then classified by BLASTN against the reference database. Gene-specific identity thresholds are applied for classification: 99.0% for 16S rRNA and 98.5% for *rpoB* at the species level, and 97.0% (16S) or 95.0% (*rpoB*) at the genus level. A final same-species consolidation step merges any clusters that received the same taxonomic assignment, summing their read counts. All read depth counts across each cluster are aggregated to produce abundance estimations for each identified taxon.

## **Detection of novel or uncharacterised species**

An important feature of FJORD is its ability to detect sequences from species not represented in the reference database. Because the pipeline generates consensus sequences by mapping to the closest available reference, a species absent from the database will still produce a clean, full-length consensus sequence provided sufficient reads are present — the consensus reflects the agreement among the reads themselves, not their match to the reference. We have documented that accurate novel consensus sequences are able to be generated within 93% identity to the closest database entry to be generated and are classified at the genus or family level according to the thresholds described above (Supplementary Table S6). This contrasts with tools that assign reads directly to database taxa, where unrepresented species are either silently misclassified as the closest database entry or discarded entirely. The accompanying consensus sequences allow users to verify any classification independently, for example by searching additional databases or by phylogenetic analysis. Preliminary results from clinical samples have confirmed the discovery of novel 16S rRNA sequences using FJORD that correspond to species-level identifications in broader databases.

## **Design considerations**

The approach described here largely prevents the undesired expansion of false positive classifications but does risk underestimating species richness in cases where several closely related species are genuinely present in the same sample. We treat this as a feature rather than a limitation, as our experience shows that clinical samples analyzed in parallel with *rpoB* amplicon sequencing — which provides higher interspecies resolution — rarely reveal such underestimation.

The pipeline produces two output files: a tab-separated table containing quantified abundances of each identified taxon with full GTDB taxonomy, classification level, and cluster membership information, and a separate multifasta file containing the corresponding consensus sequences. FJORD is available at<https://github.com/adnsvrtsn/fjord>.

## **Performance**

The most compute-extensive process within the pipeline is the X-mapper alignment, and we refer to the X-mapper paper for performance metrics. All other steps take seconds, and the full pipeline is expected to work on a standard desktop computer.

# **Acknowledgments**

Portions of the FJORD pipeline code, including embedded Python modules for sequence clustering, BLAST report parsing, and consolidation logic, were developed with assistance from Claude (Anthropic), an AI language model used as a programming aid during iterative development and testing of the pipeline.

# **References**

1.  Curry KD, Wang Q, Nute MG, Tyshaieva A, Reeves E, Soriano S, Wu Q, Graeber E, Finzer P, Mendling W, Savidge T, Villapol S, Dilthey A, Treangen TJ. Emu: species-level microbial community profiling of full-length 16S rRNA Oxford Nanopore sequencing data. Nat Methods. 2022; doi:https://doi.org/1010.1038/s41592-022-01520-4
2.  Rodríguez-Pérez H, Ciuffreda L, Flores C. NanoCLUST: a species-level analysis of 16S rRNA nanopore sequencing data. Bioinformatics. 2020; doi:https://doi.org/10.1093/bioinformatics/btaa900

3. Oxford Nanopore Technologies. EPI2ME [Software]. Oxford Nanopore Technologies Ltd. <https://epi2me.nanoporetech.com>
4.  Coster WD, Rademakers R. NanoPack2: population-scale evaluation of long-read sequencing data. Bioinformatics. 2023; doi:https://doi.org/10.1093/bioinformatics/btad311
5.  [Parks DH, Chaumeil P-A, Mussig AJ, Rinke C, Chuvochina M, Hugenholtz P. GTDB release 10: a complete and systematic taxonomy for 715 230 bacterial and 17 245 archaeal genomes. Nucleic Acids Res.](https://app.readcube.com/library/?style=Journal%20of%20Clinical%20Microbiology+%7B%22language%22:%22en-US%22%7D) 2025; doi:https://doi.org/10.1093/nar/gkaf1040

6. Lundin, D, Andersson A. SBDI Sativa curated 16S GTDB database. Linnéuniversitetet. Dataset. 2021; doi:https://doi.org/10.17044/scilifelab.14869077.v10
7.  Edgar RC. Search and clustering orders of magnitude faster than BLAST. Bioinformatics. 2010; doi:https://doi.org/10.1093/bioinformatics/btq461
8.  Shen W, Le S, Li Y, Hu F. SeqKit: A Cross-Platform and Ultrafast Toolkit for FASTA/Q File Manipulation. PLoS ONE. 2016; doi:https://doi.org/10.1371/journal.pone.0163962
9.  Li H. Minimap2: pairwise alignment for nucleotide sequences. Bioinformatics. 2018; doi:https://doi.org/10.1093/bioinformatics/bty191
10.  Danecek P, Bonfield JK, Liddle J, Marshall J, Ohan V, Pollard MO, Whitwham A, Keane T, McCarthy SA, Davies RM, Li H. Twelve years of SAMtools and BCFtools. GigaScience. 2021; doi:https://doi.org/10.1093/gigascience/giab008
11.  Gaston JM, Alm EJ, Zhang A-N. X-Mapper: fast and accurate sequence alignment via gapped x-mers. Genome Biol. 2025; doi:https://doi.org/10.1186/s13059-024-03473-7
12.  Cock PJA, Antao T, Chang JT, Chapman BA, Cox CJ, Dalke A, Friedberg I, Hamelryck T, Kauff F, Wilczynski B, Hoon MJL de. Biopython: freely available Python tools for computational molecular biology and bioinformatics. Bioinformatics. 2009; doi:https://doi.org/10.1093/bioinformatics/btp163
13.  Rognes T, Flouri T, Nichols B, Quince C, Mahé F. VSEARCH: a versatile open source tool for metagenomics. PeerJ. 2016; doi:https://doi.org/10.7717/peerj.2584
